# Resurrecting an Ancient Enzyme to Address Gene Duplication

**DOI:** 10.1371/journal.pbio.1001447

**Published:** 2012-12-11

**Authors:** Richard Robinson

**Affiliations:** Freelance Science Writer, Sherborn, Massachusetts, United States of America

**Figure:**
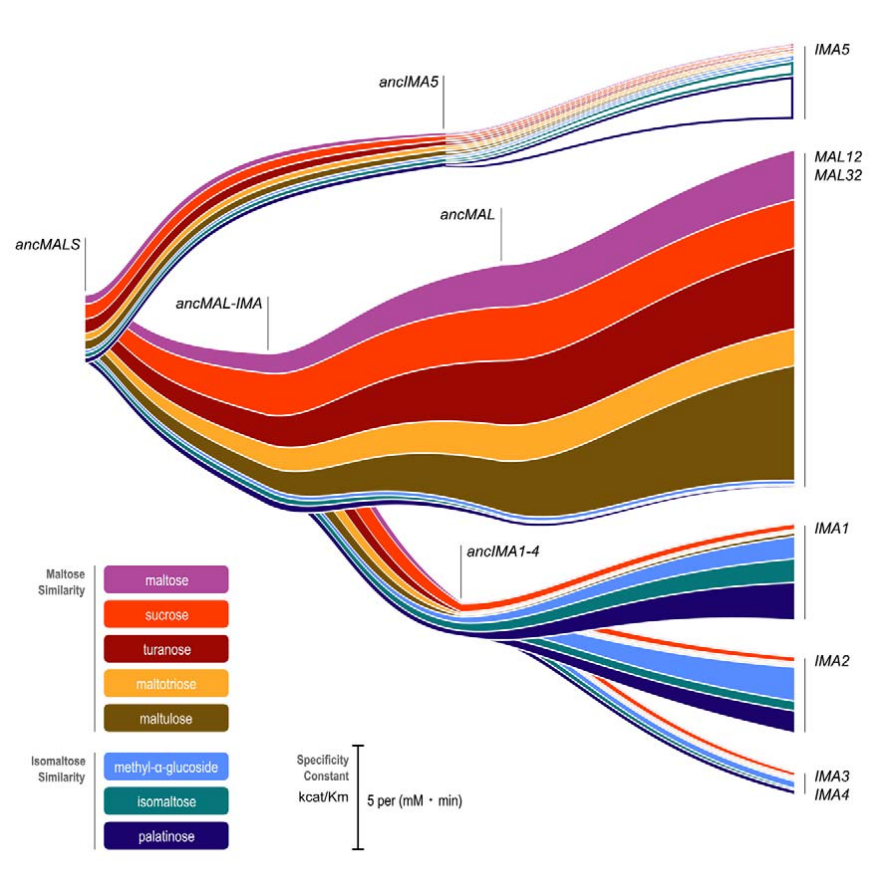
The ancestor of brewer's yeast contained one hydrolyase enzyme with broad but weak activity towards different sugars. During the course of more than 100 years of evolution, the gene encoding this enzyme was duplicated multiple times, with different copies evolving stronger and more specific activities. Image credit: Christopher Brown.

The molecular biological revolution of the past several decades has put into the hands of researchers a set of powerful tools for understanding life and its evolution. In this issue of *PLOS Biology*, Christopher Brown, Karin Voordeckers, Steven Maere, and Kevin Verstrepen put those tools to work to recreate long-vanished ancestral enzymes in vitro, testing their biochemical activity against existing enzymes to understand the consequences of one of the most common and significant evolutionary events, the duplication of an existing gene.

Gene duplication provides an organism with an extra gene copy, originally an identical one, but often not for long. Over evolutionary time, the two copies inevitably diverge in sequence, and often in function. Evolutionary theorists have described three potential protein-level consequences of a duplication event. The new gene may simply increase the “dosage," or the amount of protein created. Alternatively, as its sequence changes, it may confer a new function unavailable to the original protein, a process called neofunctionalization. Or, if the original protein had multiple functions, both old and new may evolve to specialize, each performing one of those original functions, called subfunctionalization.

The authors sought to examine how these three options have played out in a family of yeast enzymes that digest a group of sugars related to maltose. They began with the amino acid sequences of 50 maltase enzymes from a variety of species, and reconstructed the sequence of the ancestral, pre-duplication maltase from which all the others arose. They used that sequence to reverse-engineer the genetic sequence of the gene as it existed more than 100 million years ago, synthesize it from scratch, and clone it into bacteria, which then created the enzyme. They then purified the enzyme and compared its affinity and activity to enzymes from extant yeasts.

The substrate for maltase is maltose, a disaccharide comprising two glucose molecules linked in a particular way. A closely related disaccharide, isomaltose, links those same two glucoses in a slightly different way. The authors found that the ancestral maltose could split both of them, but worked much more efficiently on maltose.

Next they reconstructed the series of post-duplication sequence changes that led from the ancestral enzyme to the family of modern ones, and similarly created and tested these. The oldest of these post-duplication enzymes showed some activity for both substrates, but over time, the sequences changed to favor either maltose or isomaltose, with a significant loss of activity for the other substrate.

Within the active site, one amino acid, number 279, emerged as one of the most important determinants of whether an enzyme would favor maltose over isomaltose. Changing the amino acid at that position to increase the affinity for maltose decreased the affinity for isomaltose, they found, because the isomaltose no longer fit well into the binding pocket. A complementary change to favor isomaltose reduced the affinity of maltose. The duplication, then, allowed the yeast to evolve to maximize its utilization of both substrates, rather than abandoning one in favor of the other.

The divergence of function of these duplicated genes appears to be clear evidence for subfunctionalization. On the other hand, the authors note, the order-of-magnitude increase in digestion of isomaltose between ancestral and new enzymes might be better thought of as neofunctionalization. And their analysis of more recent duplication events showed that dosage increase appears to be at work as well, since deletion of either member of the duplicate pair led to decreased fitness.

Overall, they argue, the evidence from yeast indicates that not only may all three mechanisms be used after a duplication event, they are not necessarily separable, and all may contribute to the evolutionary path taken by a multigene family. In the case of the maltase genes, the authors suggest, the diversification that characterizes that path may have occurred at the same time as flowering plants diversified, whose sugary fruits are a key food source for many yeasts.

**Voordeckers K, Brown CA, Vanneste K, van der Zande E, Voet A, et al. (2012) Reconstruction of Ancestral Metabolic Enzymes Reveals Molecular Mechanisms Underlying Evolutionary Innovation through Gene Duplication. doi:10.1371/journal.pbio.1001446**

